# Suffix tree searcher: exploration of common substrings in large DNA sequence sets

**DOI:** 10.1186/1756-0500-7-466

**Published:** 2014-07-23

**Authors:** David Minkley, Michael J Whitney, Song-Han Lin, Marina G Barsky, Chris Kelly, Chris Upton

**Affiliations:** 1 Department of Biochemistry and Microbiology, University of Victoria, Ring Road, Victoria, BC V8W 3P6, Canada; 2 Current address: Ontario Institute for Cancer Research, MaRS Centre, Toronto, ON M5G 0A3, Canada

**Keywords:** Suffix tree, Genome, Substring, DNA sequence, STS

## Abstract

**Background:**

Large DNA sequence data sets require special bioinformatics tools to search and compare them. Such tools should be easy to use so that the data can be easily accessed by a wide array of researchers. In the past, the use of suffix trees for searching DNA sequences has been limited by a practical need to keep the trees in RAM. Newer algorithms solve this problem by using disk-based approaches. However, none of the fastest suffix tree algorithms have been implemented with a graphical user interface, preventing their incorporation into a feasible laboratory workflow.

**Results:**

Suffix Tree Searcher (STS) is designed as an easy-to-use tool to index, search, and analyze very large DNA sequence datasets. The program accommodates very large numbers of very large sequences, with aggregate size reaching tens of billions of nucleotides. The program makes use of pre-sorted persistent "building blocks" to reduce the time required to construct new trees. STS is comprised of a graphical user interface written in Java, and four C modules. All components are automatically downloaded when a web link is clicked. The underlying suffix tree data structure permits extremely fast searching for specific nucleotide strings, with wild cards or mismatches allowed. Complete tree traversals for detecting common substrings are also very fast. The graphical user interface allows the user to transition seamlessly between building, traversing, and searching the dataset.

**Conclusions:**

Thus, STS provides a new resource for the detection of substrings common to multiple DNA sequences or within a single sequence, for truly huge data sets. The re-searching of sequence hits, allowing wild card positions or mismatched nucleotides, together with the ability to rapidly retrieve large numbers of sequence hits from the DNA sequence files, provides the user with an efficient method of evaluating the similarity between nucleotide sequences by multiple alignment or use of Logos. The ability to re-use existing suffix tree pieces considerably shortens index generation time. The graphical user interface enables quick mastery of the analysis functions, easy access to the generated data, and seamless workflow integration.

## Background

Not only have Next Generation Sequencing (NGS) technologies made good on promises of cheap, high-throughput DNA sequencing, but we are poised on the brink of a 3^rd^ generation of sequencing technologies [1,2]. Among the new avenues of investigation enabled by these new technologies are: 1) a new wave of transcriptomics research [3], 2) a dramatic expansion of the catalogue of organisms for which a complete genome sequence is available, and 3) re-sequencing of multiple individual genomes for a species. Amazingly, the growth in DNA sequencing capacity and databases has been faster than the growth of computing capacity (cpu speed, or storage capabilities), which has approximately doubled every 2 years; it is therefore not surprising that the analysis of such data sets demands specialized bioinformatics tools. Equally important in the design of bioinformatics tools is the inclusion of intuitive interfaces so that researchers from both computer science and biology fields can easily use them (i.e. the software abstracts away as much underlying machinery as possible). For example, often life scientists will shy away from bioinformatics tools that are accessible only through command-line interfaces (CLIs), have steep learning curves, or don’t provide some kind of functionality for further interaction with the initial results, i.e. they prefer to use tools that function as "interactive pipelines".

In comparative genomics, common analyses include simple DNA alignments, searches for short sequence motifs, and gene content comparisons that look for the presence/absence of the genes in genomes being compared. Each has limitations, for example: 1) in long DNA sequences, rearrangements, including transpositions and inversions, can make alignments impossible, 2) gene predictions may miss annotating some genes and promoters (due to sequencing errors or poorly annotated reference genomes), 3) motif searches are too often performed using pre-existing databases of sequence patterns (no potential to find novel patterns). Therefore, to supplement these approaches in genome analysis, we have been investigating a different type of query, one that searches for short DNA sequences that are shared among a variety of long DNA sequences without the need for the sequences to be aligned. Our first tool, JaPaFi [4], searches multiple sequences (up to about 500 kb) for substrings, common to all sequences, of length *S* that have no more than *K* differences between them, however, this is limited by the length of the sequences and the diversity of the queries. After reviewing an approach akin to short-read alignment in which the "short-reads" would be extracted from a long query sequence, we considered a suffix-tree approach because this seemed likely to work better when large numbers of sequences needed comparing.

The suffix tree is a data structure that indexes a given string (DNA sequence) such that many important string operations can be performed very quickly. In particular, suffix trees provide extremely fast searching for nucleotide sub-strings, regardless of sequence size, once the trees have been constructed. While the time required for the initial construction of the suffix trees is proportional to the size of the input sequence, the constructed trees can be searched in time proportional to the length of the query sequence (i.e. search times are independent of the size of the dataset) [5]. Existing suffix tree-based search tools, such as Mummer [6], STAN [7], and Vmatch [8], are constrained by the need to maintain the constructed suffix tree(s) in RAM. Such a restriction is important because suffix trees are many times the size of the input sequence, meaning that, for example, even a single mammalian genome could generate suffix trees that exceed the memory capacity of the average desktop computer. Faster suffix tree tools, such as TRELLIS + [9], DiGeST [10] (developed by MGB), and ERa [11] solve the tree size limitation, but are accessible only through the command line, and provide little to no pipelining features, thus decreasing their appeal to life scientists.

Our tool, Suffix Tree Searcher (STS) allows the analysis of large numbers of unaligned long DNA sequences through the application of disk-based partitioned suffix trees (based on MGB’s DiGeST). In the development of STS, we have continued with the goal of providing powerful software for the bench scientist with minimal computer science experience. Accordingly, the program is accessed through a Java Web Start link on a web page, which automatically installs or updates the program files for the user. Interaction is conducted through a graphical user interface (GUI) that allows the user to construct indexes of the input sequences and quickly perform a variety of queries on the sequence data. Results are presented to the user in tabular form, can be sorted based on multiple criteria, and are easily integrated into subsequent queries with a mouse click, providing for a natural analysis workflow (see case studies). In addition, since the initial construction of suffix trees is computationally expensive, STS allows the user to load previously constructed suffix trees for analysis. Thus, once the suffix tree forest has been constructed for a given data set, future analyses can be run much more quickly by skipping the most time-consuming step.

## Methods

### Organization of the STS program

**Figure 1:**
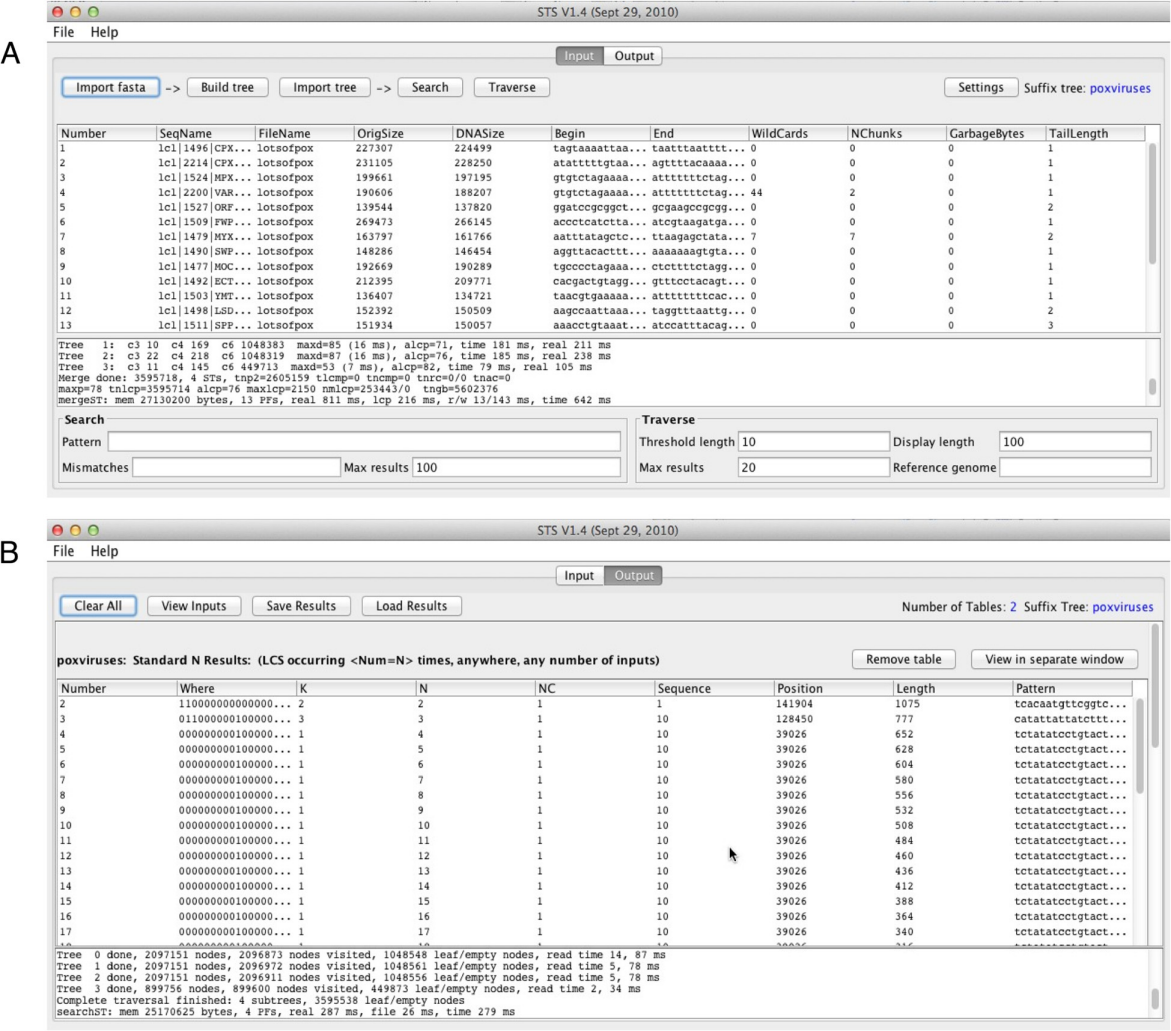
**STS graphical user interface.** Panel **A**: Input window. Panel **B**: Output window.

STS is comprised of four separate C modules, *fastaST*, *ssortST*, *mergeST*, and *searchST*, which implement the functionality, and a Java GUI. When the GUI is launched via a Java Web Start link, the C programs are automatically downloaded, and installed in a directory called *exe* within the user’s home directory. The programs and GUI are updated, if needed, each time STS is initiated. The GUI (Figure 1) includes a text area that is used by the C programs for reporting execution progress (and errors). It is important to note that the underlying C programs are hidden by the GUI, so that in normal usage the user does not require any knowledge of their implementation. Currently, STS runs on Linux and OS X operating systems. Users should also be aware that STS does not automatically search the reverse complement of sequences.

In order to analyse a data set, the sequence(s) must first be *imported*, and then the trees must be *built*. Behind the scenes, each GUI action invokes one or more of the C modules to do the work. Specifically, the *import* command invokes the *fastaST* module, and the *build tree* command invokes the *ssortST* and *mergeST* modules in order, while *searches* and *traversals* are conducted through the *searchST* module.

### fastaST

This module reads one or more FASTA formatted files, each containing one or more of the DNA sequences to be analyzed. *fastaST* is invoked when the user clicks the *Import fasta* button in the *Input* window of the STS GUI. All characters except A, C, G, and T are ignored (Figure 1). However, non-ACTG characters are counted and reported to the user. Imported sequences are associated with a unique number that is displayed in the *Input* window along with sequence names, lengths, segments from the beginning and ends of the sequences, and whether the sequence contained any ‘N’ characters.

### ssortST

This module uses in-memory "lightweight" suffix sorting routines to sort individual or multiple nucleotide sequences so as to maximize use of computer memory and minimize the disk-intensive merge phase that follows (implemented by *mergeST*). We use Mori’s suffix sorter for this task [12], which has a memory usage of 5*n* bytes, where *n* is the sum size of the sorted input sequences. The sorter is modified to handle sorting of multiple sequences, and also to calculate the longest common prefix (LCP) values of adjacent suffixes, using an algorithm due to Manzini [13]. The LCPs calculated here help the subsequent merge to run much faster when the data is repetitive (e.g. due to closely related sequences).

### mergeST

This module is a modified version of the DiGeST algorithm, and forms the core of the suffix merge algorithm mentioned above. Here, chunks of the sorted sequences are merged together into a large lexicographically partitioned suffix tree. Each partition is of a size that fits the cache capacity of current computers, but can be adjusted by the user, like most other STS parameters. One drawback of previous suffix tree construction algorithms was the requirement for the constructed suffix trees to fit into main memory. However, disk-based approaches such as the DiGeST algorithm require only that the input sequence fit into main memory. In addition, since DiGeST compresses the input sequence to use only 2 bits per nucleotide, the amount of main memory, which can be virtual memory, used is actually 1/4 the size of a normal FASTA sequence encoded using 8 bits per character (e.g. UTF-8 or ASCII).

The mergeST function offers speed improvements over the original DiGeST algorithm when dealing with highly similar sequences, such as those resulting from a comparison of the two human sex chromosomes. The merge phase of DiGeST requires LCP values between lexicographically adjacent suffixes when adding these strings to a growing suffix tree. In DiGeST, these LCPs are generally calculated using the first 32 nucleotide bases of each of the two suffixes; these 32 bases are stored in memory for each suffix. When two suffixes are sufficiently similar (sharing at least their first 32 bases), the merge phase of DiGeST must utilize a time-consuming disk read operation to retrieve subsequent nucleotides and calculate the LCP. The mergeST function of STS utilizes LCP values which were previously calculated by the ssortST function when disk I/O was already taking place. As a result, STS receives a modest increase in speed when processing very similar sequences.

### searchST

The purpose of this module is to examine the suffix trees and provide result tables to the user. Results are categorized as *search* results, or *traversal* results (Figure 1). *Searches* can either be for an exact match to a provided pattern, or for matches with some allowed number of base substitutions from the pattern. Query patterns may contain wildcard characters (‘*’) that will match any nucleotide. Exact matches to patterns containing no wildcards are found by following a single path from the root of a suffix tree and are performed very quickly in time proportional to the length of the query sequence. Searches for patterns containing wildcards or allowing some number of dissimilar bases between the query and a match will analyze all permitted paths in the suffix tree. For example, if a single wildcard exists in a query pattern, up to four paths of the suffix tree (corresponding to the four standard nucleotide bases) will be explored. As a result, search time has the potential to increase exponentially with respect to the number of wildcards added or non-matching bases permitted.

*Traversal* results require visiting the entire suffix tree (a bottom-up tree traversal), which can take a significant amount of time for very large trees. Much of this is file access time, since the suffix tree is disk-based. Traversal results report on the number of times patterns of various lengths occur in the suffix tree dataset. An example of a traversal result set is a report of the longest exact matches among a set of inputs for each *n*, where *n* is the number of times the exact sequence is found in all sequences in the dataset.

### Re-use of existing data

Since the trees generated from large data sets take significant time to *build*, STS provides the option to save and re-load trees using the *Import tree* button and to save and re-load individual *Results* sets using the respective buttons in the ‘*Output*’ view. STS does not support the ability to incrementally add sequences to previously created suffix trees.

### User interface

The STS Java GUI consists of a single window with tabs to navigate between the *Input* and *Output* views. The user toggles between tabs using buttons at the top of the window. The *Input* screen is used to select sequence files for analysis, initiate tree *building*, initiate the tree *traversal* analysis for collecting common subsequence data, and run tree *searches* for specific nucleotide strings. Results are presented in panels in the *Output* screen, with new panels added for each additional search. An important feature of the *Results* window is the ability to sort the results by the various attributes in the different columns; multiple sorting rules can be combined, and individual results sets can be exported into their own windows.

Sequences in the *Results* window, corresponding to rows of the tables, can be used directly in subsequent searches through a right-mouse-click menu. We have found this to be a very useful feature, permitting the user to quickly and intuitively gather more exact data about a particular substring when needed. This function also works for a set of substrings, as STS supports searches for multiple substrings at once. The column sorting facilities enable the user to assemble and then select the table rows of interest for further analysis. The menu also permits users to view one or more selected substrings in the context of their surrounding sequences.

### Tree traversals

Tree *traversals* create a complete summary of the relationships among all of the strings of nucleotides to identify common patterns, and require examination of all nodes in the suffix trees. We found this function to have a variety of research applications, which are discussed below.

A basic tree *traversal* provides results in two tables. The *Standard N Results* table provides information about the different longest common substrings (LCS) that occur exactly *n* times, for *n* = 2,3,4… without regard to which input sequences they occur in. The single LCS that occurs in exactly *k* inputs, for *k* = 2,3,4… is shown in the *Standard K Results* table. The *traversal* analyses are modified using parameters accessed from the *Settings* button, and the display of results is controlled using the *Traverse* section of the *Input* window (Table 1).

**Table 1:** Parameters for tree traversals and display of traversal results

	**Setting**	**Explanation**
***Traversal settings***	LCS occurrences	Gather LCSs occurring for *n* = 2, … up to the number specified by this setting
	LCS inputs	Gather the single LCS in *k* = 2, … input sequences up to the number specified by this setting
	Number of occurrences	For each number *n* specified, fetch the *p* longest LCSs occurring exactly *n* times, regardless of input sequence
	Number of inputs	For each number *n* specified, fetch the *p* longest LCSs occurring at least once
	Sets	Fetch the *p* best results for each set of input sequences as specified by this setting. Sets are specified with braces. e.g. {2}{2,3}{2,3,4}{2,4}{5,6}
	All singles	Gather a separate result set for each input sequence.
	All pairs	Gather a separate result set for each possible pair of input sequences
***Traversal results display***	Threshold length	Common substrings must be of at least this length to show up in result sets
	Display length	Display LCSs only up to and including this length in the result sets
	Max results	For the non-standard result sets, restrict table size to this many rows
	Reference genome	Gather result sets only for queries involving this input sequence

### Searching trees

One of the most important features of STS, and of suffix trees in general, is that searching for specific sequences is extremely fast. With the exception of selecting a query sequence directly from the *Results* window, all other searches are initiated from the *Input* window, which contains a search box that is used to specify a variety of queries (see Table 2 for query options). Multiple queries can be searched in one run by entering the search terms for each individual query sequence into the search box, separated by semicolons. In addition, the user can specify the maximum number of results desired, as well as the number of mismatches and the location of any wildcards. A mismatch allows a difference between the search sequence and the dataset sequences at any position in the search sequence, while a wildcard is a difference at a single, specific position within the search sequence. Wildcards are represented in the search box with a ‘*’, while the number of mismatches is specified in a box adjacent to the search box. The *Output* window displays the list of hits, providing the names of sequences and hit position. Importantly, the user can retrieve and export FASTA files of the actual hit sequences by simply selecting the required rows of hits, using a right-mouse-click, and selecting the *Export Common Substring(s)* option.

**Table 2:** Example input formats for search sequences

**Input pattern**	**Interpretation**	**Comment**
acgcgaatccgt	Search all input sequences for pattern	Multiple patterns are separated with a ";"
ac*cg*atccgt	Search all input sequences for pattern. "*" represents a wildcard, which matches all 4 nucleotides	Multiple patterns are separated with a ";"
"2, 12961, 12"	Search input 2 for a 12 nt pattern beginning at position 12961	Multiple patterns are separated with a ";". Tandem repeats of the pattern may be matched if they exist.

## Results and discussion

### Analysis of randomly generated sequences

In order to evaluate the performance of STS (speed, maximum sequence lengths, maximum numbers of sequences) in a way that was not biased by similarity between the particular input sequences, sets of randomly-generated FASTA-formatted DNA sequences were used as inputs for testing. These and all other tests mentioned in this paper were conducted on an early 2008 iMac with 4GB of DDR2 RAM, a 2.8 Ghz Intel Core2 Duo processor and 6 MB of shared L2 cache. Due to the significant amount of disk space required to store on-disk suffix trees for large datasets, an external USB 2.0 hard drive was used in the creation and testing of two datasets: the "10,000 randomly generated 1Mbp sequences" dataset and the human genome dataset. The disk data transfer speed for these tests is potentially throttled by the USB 2.0 connection. It is important to note that the trees for any particular data set need only be *built* once, after which point they reside on the hard disk, and can later be reloaded in seconds at the user’s discretion.

The maximum number of 10,000 nt sequences that could feasibly be analyzed in one day was on the order of 100,000 sequences, giving an aggregate size of 1,000,000,000 nt (Table 3). The maximum length of the individual sequences that could be analyzed in a single day (tested as a set of 10,000 sequences) was on the order of 1,000,000 nt, giving an aggregate size of 10,000,000,000 nt (Table 4). *Traversals* always took longer than *build* times for data sets with a total size of 100,000,000 nt or more. The number of sequences in a data set had a greater affect on *traversal* times than did the lengths of individual sequences for a given total number of nucleotides. However, this difference was only appreciable when the total size of the data set was on the order of 1,000,000,000 nt or greater. Search times for all test datasets were less than one second, and all used approximately 25 Mb of memory.

**Table 3:** Sequence summary and resource usage for tree construction and traversal of test datasets

		***Tree construction***				***Full traversal***	
**Dataset**	**Size (Mbp)**	**Time**^**1**^	**Rate (kbp/s)**	**Peak memory (Gbp)**	**Disk usage**	**Time**	**Peak memory (Mbp)**
10 random 10 kbp seqs	0.1	154 ms	649.4	1.72	4.0 Mb	21 ms	2.3
100 random 10 kbp seqs	1.0	733 ms	1,364.3	1.72	39.7 Mb	150 ms	22.9
1,000 random 10 kbp seqs	10.0	9.2 s	1,084.9	1.72	397.1 Mb	3.8 s	24.0
10,000 random 10 kbp seqs	100.0	1 m 56 s	861.3	1.72	3.9 Gb	5 m 10s	24.1
100,000 random 10 kbp seqs	1,000.0	29 m 52 s	558.1	1.72	38.8 Gb	7 h 23 m	25.2
10,000 random 100 bp seqs	1.0	6.0 s	166.4	1.72	111.6 Mb	1.7 s	22.0
10,000 random 1 kbp seqs	10.0	13.7 s	732.1	1.72	459.7 Mb	25.5 s	24.1
10,000 random 100 kbp seqs	1,000.0	25 m 42 s	648.6	1.72	38.5 Gb	51 m 22 s	24.3
10,000 random 1 Mbp seqs^2^	10,000.0	12 h 29 m	222.5	1.72	384.7 Gb	9 h 18 m	24.0
62 *E. coli* genomes	310.5	21 m 55 s	235.7	1.72	11.9 Gb	2 m 33 s	24.0
62 random seqs w/ *E. coli* lengths	310.5	7 m 3 s	734.0	1.72	11.9 Gb	2 m 56 s	24.0
4 Chlorella virus genomes	0.9	815 ms	1,074.5	1.72	41.8 Mb	203 ms	24.0
Human genome (hg38)^2^	3,209.3	3 h 58 m	223.7	2.07	117.2 Gb	45 m 33 s	24.1

^1^Tree construction time includes time to both *Import* sequences and *Build* the suffix index.

^2^The "10,000 random 1 Mbp" and human genome datasets utilized an external USB2.0 hard drive.

**Table 4:** Effect of mismatches and wild cards on search times

**Sequence**	**Mismatches**	**Hits**	**Search time**
*agtcagtactgga*	0	2	461 ms
	1	61	1.4 s
	2	1075	1.8 s
	3	12543	6.8 s
	4	98684	18.9 s
agtcagtac*gga	0	4	28 ms
	1	234	1.4 s
	2	3757	2.5 s
	3	39076	8.6 s
	4	277422	33.0 s
agt*agtac*gga	0	30	76 ms
	1	807	5.9 s
	2	12777	6.4 s
	3	118420	17.4 s
	4	754861	84.3 s
agt*agtac*g*a	0	93	94 ms
	1	2923	32.7 s
	2	41372	1 m 0 s
	3	350292	1 m 27 s
	4	19708731	2 m 31 s

Searches were performed on trees constructed from a dataset of 10,000 randomly-generated 10 kbp sequences.

In order to test the effects of mismatches and wildcards on search times, a specific 13 nucleotide sequence was searched for in a tree of 10,000 randomly generated sequences of length 10,000 nt, specifying varying numbers of mismatches and wildcards (Table 4). Mismatches affect search times more than wildcards because mismatches can occur at any position in the query sequence whereas wildcards are restricted to specific positions.

### Analysis of small genomes

While tests using randomly generated sequences are useful in providing a general picture of performance relative to data set size, they cannot accurately predict performance for real sequences that have significant similarity to each other. Therefore to provide a more realistic test case, STS was used to analyze a data set containing every *E. coli* genome available from GenBank [14] (62 genomes, June 2014), ranging in size from 3.9 to 5.7 million nt (Table 3). The *traversal* time for 62 genomes was approximately 2.5 minutes, whereas the *construction* time (i*mport + build)* was approximately 22 minutes. In contrast, a data set of 62 randomly generated sequences with the same number of non-N bases as the *E. coli* data was *constructed* in seven minutes and *traversed* in approximately 3 minutes. The 3-fold discrepancy between the *build* time for the *E. coli* and randomly-generated sequences illustrates the need to supplement testing on randomly generated sequences with testing on real data sets.

As noted above, *traversals* find, among other results, the longest sequences that are present *n* times in the data set. Once these or any other sequences have been identified in the results table, a right-click menu provides the ability to obtain more specific information, such as identifying the inputs that contain a selected motif, or highlighting a motif in the context of the surrounding sequence. Furthermore, any sequence presented in the program window can be copied for pasting into other sequence analysis programs, or other parts of STS, to provide a smooth transition between different analyses.

For example, from *traversing* a single *E. coli* genome [GenBank:FN554766.1], the 3 longest sequences found *n* times in the genome, where *n* = 2, 3, and 4, were 5092 nt (*n* = 2), 1292 (*n* = 3) and 818 nt (*n* = 4). After a pattern search of the 5092 nt sequence revealed the two locations in the genome, further investigation indicated that these regions corresponded to 16S and 23S rRNA, and these results were confirmed with BLASTN [15]. Similarly, the 1292 nt and 818 nt patterns were found in 3 and 4 23S rRNAs throughout the genome, respectively. Although, as used above, the LCS approach to finding common motifs is inherently superficial – of the 7 listed 23S rRNA in this *E. coli* genome, only up to 4 were identified in this process – it provides a quick overview of repeated regions within a single genome.

To further demonstrate the investigative utility of the program, STS was used to examine the similarities among 4 Chlorella viruses: ATCV-1, MT325, NY2A, and PBCV-1 (sequences obtained from Greengene [16]). Both PBCV-1 and NY2A infect *Chlorella* NC64A, while MT325 and ATCV-1 each infect different *Chlorella* hosts, respectively. Therefore, it was hypothesized that PBCV-1 and NY2A would have more motifs in common than either MT325 or ATCV-1 [17].

Focusing first on the patterns present in all 4 genomes, yielded by a *traversal* (Table 3), we visualized their locations in the genome using VGO [18], and found that these motifs were most common between genes, suggesting that they were promoters or other regulatory motifs. A search for the most frequently occurring 10 nt pattern common to all 4 genomes, allowing for one mismatch, resulted in 255 hits, which were then exported to a single FASTA file through the right-click menu. The FASTA file was used to draw a logo (figure not shown), resulting in the consensus pattern TTTGTCGATA, a motif consistent with a putative promoter previously suggested by Fitzgerald *et al.*[17]. In addition, Fitzgerald *et al.* identified 3 other potential promoters, two of which were found in our STS results. The fourth putative promoter, ARNTTAANA, was likely not found due to the multiple wildcards (‘R’ and ‘N’) in the sequence. It should be noted that STS only indexes and searches the DNA strands given in the input and search sequences, respectively (i.e. the program does not convert any sequence to its reverse-complement). Accordingly, to find a particular pattern on both strands, the reverse complement of the pattern should be searched; however, restricting the search to one strand at a time greatly simplifies the evaluation of the output for the user.

Closer analysis of the other results revealed the presence of DNA patterns encoding PAPK repeats in certain proteins. This finding has been previously reported by Onimatsu *et al.*[19]. These two motifs were found at much higher frequency in NY2A and PBCV1, supporting our original hypothesis based on similarity of host discrimination. As shown above, when analyzing multiple genomes, STS allows for a simple and quick overview of all types of common motifs between sequences through *traversals*, without being restricted by the size or number of the inputs. This example also demonstrates how STS facilitates fast pattern searching with mismatches, sorting of results based on location, and easy exporting of results for use with other tools. The value of STS in this example is that these viruses, although related, are so different as to make the generation of a meaningful multiple sequence alignment almost impossible.

### Analysis of mammalian genomes

With the advent of the "$1000 human genome" on the horizon and the decreasing costs of sequencing options, working with data sets as large as the human genome may soon become a much more frequent occurrence. To simulate such a scenario, the human genome was analysed with STS (Table 3). *Importing* the FASTA files (containing 3.2 billion nt), sorting the individual chromosomes, and building the tree, which occupied 117.2 GB of disk space, took approximately four hours total. Tree *traversals* required approximately 45 minutes, depending on the complexity of the search. The longest sequence found to occur twice in the human genome was a 2,449,175 nt segment present on the X and Y chromosomes (in the pseudoautosomal region).

The LCS found in all 24 chromosomes was 294 nt long and was present a total of 108 times; finding the locations of these 108 sequences took approximately 5 seconds and revealed that they were not significantly clustered (data not shown). This LCS was found to be a member of the *Alu* family, highlighting how STS could be used, in this case, to very quickly find any particular subset of *Alu* sequences; use of wild cards would also allow greater variation in the sequences discovered. The analysis could be modified to find the 500 most frequently occurring sub-sequences common to all chromosomes by setting the *Number of Inputs* to 24 in the *Traverse Settings.* It would then be simple to retrieve these sequences for further analysis and comparison.

Since most of the time required by *traversals* is spent reading the sub-tree files from disk, it is most efficient to perform fewer, but more comprehensive searches, e.g. by selecting *All singles* from the *Sets* section of the *Traverse Settings*, which effectively generates *traversal* data for the individual chromosomes. By decreasing the number of searches, the same amount of information can be yielded, with reduced disk I/O - and thus time - cost.

*Traversal* times can be significantly decreased by de-selecting the *LCS inputs* setting, which removes the requirement for STS to calculate the LCSs that occur in exactly k inputs (the second table produced by a *traversal* with default settings). In order to produce this table, the searchST phase of STS must associate each substring in the suffix tree with all of the input sequences it is present in. Determining these associations for each node in the suffix tree results in a significant slowdown that is more pronounced for data sets with larger numbers of sequences. For example, a *traversal* of a tree of 1,000,000 randomly generated 1,000 nt sequences took 69.8 hours with default parameters, but de-selection of the *LCS inputs* setting reduced this time to 12.7 minutes.

### Improvements over other tools

STS was designed with the goal of giving life sciences researchers access to the powerful sequence analysis capabilities provided by suffix trees, while maintaining a minimum of effort on the part of the researcher. Existing suffix tree-based analysis tools, such as MUMmer and STAN require that the suffix trees, which are often many times the size of the input sequence, fit entirely into RAM during construction; this places severe limits on the total size of the input sequences. In comparison, STS uses a disk-based approach to suffix tree construction, requiring only that a compressed copy of the input sequence fit in main memory. As a result, STS requires only 2 bits of memory per nucleotide, compared to the minimum 12.5 bytes (100 bits) per nucleotide required by MUMmer.

Although disk-based suffix tree construction algorithms exist that are faster than the one used by STS (e.g. B2ST [20], and ERa), the existing implementations of these algorithms lack analytic capabilities such as LCS traversals, require manual compilation, and are accessible only through CLIs, making usage more difficult for the non-technical user. In comparison, STS provides a GUI, requires no additional set up following download, and adds a variety of searching and analysis features, giving researchers who have little computer science knowledge an intuitive way to interact with some of the most powerful suffix tree algorithms available. Users have only to assemble one or more FASTA-formatted files of their sequences to be able to search and manipulate very large sequence data sets on a desktop computer. The STS interface also provides easy access to the results, in a window with cut/paste features, which enables users to quickly export data into other sequence analysis programs.

### Future improvements

A number of areas have been targeted for improvement in the next version of STS. One present limitation is the fact that two files are presently created on disk for each input sequence – one binary file, and one text file. Although we encountered no difficulties working with very large data sets on a basic desktop machine, we did find that using a dedicated hard drive was useful for large datasets to 1) avoid system problems if STS filled up the drive, and 2) allow rapid reformatting of the drive in order to quickly delete the millions of files that accrued during testing, analysis and debugging. We are currently exploring techniques for decreasing the amount of disk space occupied by the suffix trees.

Another current limitation is that the *traverse* function may identify certain repeating sequences multiple times at different lengths. For example, a trinucleotide CAG repeat might also be reported as a hexanucleotide CAGCAG repeat if no longer LCS exists for the given *n-value*. However, the *traversal* output reports the number of sequences present for a given *n* as *NC,* and these sequences can be identified by customizing the *number of occurrences traverse setting*. We hope to eliminate this limitation in future releases.

An additional limitation is presented by the large number of ‘N’s in mammalian genomes. Converting large blocks of Ns to wildcards could cause misleading matches to other LCSs during *traversals*, potentially eclipsing any given LCS for a value of *n* (where *n* is the number of times that the LCS occurs in the data set), and otherwise skewing results. In order to avoid this problem, STS ignores ‘N’ characters in the input sequences. However, the numbers of individual ‘N’s and blocks of ‘N’s in each input are still counted and displayed in the *Input* window. While this approach potentially generates a small number of new sub-sequences (e.g. ACGT from ACNGT, instead of AC*GT), the method has been found by Barsky *et al.*[10] to cause no problems when using the generated suffix trees .

## Conclusion

STS provides a new method to summarize common sequence strings and perform a variety of search functions on large DNA data sets without the need for these sequences to be aligned. Although suffix trees have been previously used in a variety of bioinformatics applications, our implementation of disk-based suffix trees allows both the construction and analysis of indices from very large DNA data sets. The program is web-delivered, platform-independent, and accessed via a user-friendly GUI, allowing for a more intuitive workflow, and potential integration into a genome browser.

### Availability and requirements

**Project name:** Suffix Tree Searcher (STS).

**Project home page:**http://athena.bioc.uvic.ca/virology-ca-tools/sts/. A Quick Start Guide and Manual are available.

**Operating System(s):** Mac OS X (Intel), Linux.

**Programming language:** C, Java.

**Other requirements:** Java 1.6.

**License:** GNU General Public License.

**Any restrictions to use by non-academics:** None.

## Competing interests

The authors declare that they have no competing interests.

## Authors’ contribution

MJW was an initial designer and programmer of the STS tool, which was based on prior work of MGB; DM continued with programming and designed, implemented the routines for searching with mismatches and wild cards, and performed testing and debugging; SHL implemented the Java interface; CK helped test the tool; CU contributed ideas for features and display requirements, tested the program and wrote the manuscript with DM, MJW and CK. All authors have read and approved the manuscript.
